# Comparative Outcomes of Minimally Invasive Versus Open Pancreatoduodenectomy in Distal Cholangiocarcinoma: A Systematic Review and Meta-Analysis

**DOI:** 10.7759/cureus.59404

**Published:** 2024-04-30

**Authors:** Sabrina Domene, Manuel Quiroz Flores, Daniela Fulginiti, Karem D Thomas Garcia, Nathnael Abera Woldehana, Karleska M Nunez Jimenez, Víctor M Lagos Herrarte, Jose A Guevara Benavides, Carlos R Alegría Perdomo, Cesar Estrella-Gaibor, Victor Sebastian Arruarana, Marily Martinez Ramirez

**Affiliations:** 1 General Practice, Universidad Nacional de Mar del Plata, Mar del Plata, ARG; 2 Surgery, Universidad de Antofagasta, Antofagasta, CHL; 3 General Practice, Pontifical Catholic University of Argentina, Buenos Aires, ARG; 4 General Practice, Universidad de Oriente, Anzoátegui, VEN; 5 General Practice, Johns Hopkins Bloomberg School of Public Health, Baltimore, USA; 6 General Practice, Universidad Católica del Trópico Seco, Nicaragua, NIC; 7 General Practice, Universidad de Carabobo, Valencia, VEN; 8 General Practice, Universidad Autónoma de Honduras, Tegucigalpa, HND; 9 General Surgery, Ministry of Public Health, Hospital Esmeraldas sur Delfina Torres de Concha, Quito, ECU; 10 Internal Medicine, Brookdale University Hospital Medical Center, New York, USA; 11 Internal Medicine, Universidad Nacional Autonoma de Mexico, Mexico City, MEX

**Keywords:** laparoscopic pancreaduodenectomy, whipple procedure, traditional pancreatoduodenectomy, robot-assisted pancreatoduodenectomy, cholangiocarcinoma

## Abstract

Pancreatoduodenectomy, the primary surgical strategy for managing cholangiocarcinoma, is executed via two distinct methodologies, namely minimally invasive pancreatoduodenectomy (MIPD) and open pancreatoduodenectomy (OPD). The selection between these surgical options is critical, as it directly influences patient outcomes, encompassing both short-term recovery metrics and long-term survival rates. Despite the clinical significance of these procedures, there exists a notable void in the literature regarding a comprehensive comparison of MIPD and OPD, particularly in assessing their respective efficacies and complications. This lack of detailed comparative analysis has left a gap in evidence-based guidance for clinicians faced with the decision of choosing the most appropriate surgical approach for their patients. The absence of robust data comparing the two techniques underscores the necessity for a meta-analysis that rigorously examines and contrasts the outcomes associated with MIPD and OPD. By drawing upon a wide array of international studies, this research aims to shed light on the advantages and potential drawbacks of each method, thereby providing a more informed basis for surgical decision-making in the treatment of cholangiocarcinoma.

## Introduction and background

Distal cholangiocarcinoma (dCCA) is a malignant neoplasm located in the last portion of the biliary tract, specifically from the junction of the cystic and hepatic ducts to the ampulla of Vater [1]. This neoplasm is an aggressive form of epithelial origin, representing approximately 30% of all cholangiocarcinomas [2]. The incidence of dCCA varies from 20% to 40% [3], with a higher prevalence reported in certain regions, such as Southeast Asia. The etiology of dCCA is multifactorial, involving risk factors such as chronic inflammation, biliary tract diseases, primary sclerosing cholangitis, and parasitic infections, particularly in areas endemic for liver flukes [4,5]. Other factors, such as intracellular communication with extracellular vesicles, are being revealed [6,7].

Pancreatoduodenectomy (PD) is the surgical procedure of choice for treating dCCA, which can be performed with an open pancreatoduodenectomy (OPD) or minimally invasive pancreatoduodenectomy (MIPD), including laparoscopic pancreatoduodenectomy (LPD) or robotic pancreatoduodenectomy (RPD). There is insufficient evidence to support chemotherapy or radiotherapy as a treatment of choice [8]. Although medicine has advanced in recent years in terms of mortality, the survival rate of patients with dCCA remains low, reaching only 5% at five years [9]. Lower survival and recurrence rates have been associated with multiple features, including pancreatic, perineural, and lymphatic invasion, lymph node metastasis, and pathologic tumor stage ≥T35 [10]. Postoperative morbidity rates are also high, ranging between 30% and 50%. The main factors contributing to this are infections, fistulas, and delayed gastric emptying, which can occur in OPD and MIPD [9]. The disease's aggressive clinical characteristics and surgical complications create a complex scenario, as evidenced by the mortality and morbidity rates.

Numerous studies have shown evidence supporting the advantages of MIPD over OPD, including shorter hospitalization and faster recovery [8,11-13]. However, other studies have found no advantage of open surgery over laparoscopic surgery, such as oncological success or mortality rate differences [14-16]. Intraoperative and postoperative complications remained the same [16]. Several unclear factors remain, which are crucial in the decision-making analysis of an intervention, such as blood loss during the procedure, patient survival rates, and possible postoperative complications. The lack of consideration of these elements leaves a knowledge gap that our study aims to address.

Distal cholangiocarcinoma is a very aggressive disease with a low survival rate [9]. The diversity of available evidence highlights the need for a systematic review to analyze the existing information and arrive at a conclusion. Healthcare professionals must choose the most appropriate treatment to improve quality of life and survival, so it is necessary to reach common ground on the diversity of literature that has been found. Therefore, in this review, we propose to understand the differences between laparoscopic and open surgery in patients with dCCA and which of these is more beneficial for patients in terms of short- and long-term goals.

## Review

Methods

The present study employed the Preferred Reporting Items for Systematic Review and Meta-Analysis (PRISMA) 2020 guidelines to conduct a comprehensive systematic review [17,18]. The protocol for this review has been registered in the Prospective Register of Systematic Reviews (PROSPERO) database (no. CRD42024507019). We searched PubMed (Table 1), Scopus (Table 2), Web of Science (Table 3), Embase (Table 4), and Cochrane (Table 5) using medical subject headings (MeSH) terms and free text terms on 01/24/2024. 

**Table 1:** MeSH terms used to search PubMed MeSH: Medical subject headings

Search terms	Results
((Distal Cholangiocarcinoma [MeSH]) OR (Bile Duct Neoplasms [MeSH]) OR (cholangiocarcinoma [Title/Abstract]) OR (bile duct cancer [Title/Abstract])) AND ((Minimally Invasive Pancreatoduodenectomy [Title/Abstract]) OR (Laparoscopic Pancreatoduodenectomy [Title/Abstract]) OR (Robot-Assisted Pancreatoduodenectomy [Title/Abstract]) OR (Laparoscopic Whipple Procedure [Title/Abstract]) OR (Robotic Whipple Procedure [Title/Abstract] OR (Open Pancreatoduodenectomy [Title/Abstract]) OR (Traditional Pancreatoduodenectomy [Title/Abstract]) OR (Open Whipple Procedure [Title/Abstract]))	324

**Table 2:** Terms used to search Scopus

Search terms	Results
ALL (distal AND cholangiocarcinoma OR bile AND duct AND neoplasms) AND (minimally AND invasive AND pancreatoduodenectomy) AND (open AND pancreatoduodenectomy)	207

**Table 3:** Search terms used for Web of Science

Search terms	Results
ALL=(Distal Cholangiocarcinoma)	988
ALL=(Cholangiocarcinoma)	23751
ALL=(Bile duct neoplasms)	2314
ALL=(Bile duct cancer)	10235
ALL=(Minimally Invasive Pancreatoduodenectomy)	207
ALL=(Laparoscopic Pancreatoduodenectomy)	334
ALL=(Robot-Assisted Pancreatoduodenectomy)	43
ALL=(Laparoscopic Whipple Procedure )	134
ALL=(Robotic Whipple Procedure )	43
ALL=(Open Pancreatoduodenectomy)	409
ALL=(Traditional Pancreatoduodenectomy)	36
ALL=(Open Whipple Procedure)	143
#1 OR #2 OR #3 OR #4	31205
#5 OR #6 OR #7 OR #8 OR #9 OR #10 OR #11 OR #12	812
#13 AND #14	38

**Table 4:** Terms used to search EMBASE

Search terms	Results
#1 ‘distal cholangiocarcinoma'	626
#2 ‘cholangiocarcinoma'	30203
#3 ‘bile duct neoplasms'	342
#4 'bile duct cancer'	7517
#5 ‘minimally invasive pancreatoduodenectomy'	99
#6 ‘laparoscopic pancreatoduodenectomy'	262
#7 ‘robot-assisted pancreatoduodenectomy'	30
#8 ‘laparoscopic whipple procedure'	30
#9 ‘robotic whipple procedure'	17
#10 'open pancreatoduodenectomy'	189
#11 ‘traditional pancreatoduodenectomy'	0
#12 'open whipple procedure'	9
#13 ‘distal cholangiocarcinoma' OR 'cholangiocarcinoma' OR 'bile duct neoplasms' OR 'bile duct cancer'	36153
#14 minimally invasive pancreatoduodenectomy' OR 'laparoscopic pancreatoduodenectomy' OR 'robot-assisted pancreatoduodenectomy' OR 'laparoscopic whipple procedure' OR 'robotic whipple procedure' OR 'open pancreatoduodenectomy' OR 'traditional pancreatoduodenectomy' OR 'open whipple procedure'	524
#15 ('distal cholangiocarcinoma' OR 'cholangiocarcinoma' OR 'bile duct neoplasms' OR 'bile duct cancer') AND ('minimally invasive pancreatoduodenectomy' OR 'laparoscopic pancreatoduodenectomy' OR 'robot-assisted pancreatoduodenectomy' OR 'laparoscopic whipple procedure' OR 'robotic whipple procedure' OR 'open pancreatoduodenectomy' OR 'traditional pancreatoduodenectomy' OR 'open whipple procedure')	25

**Table 5:** Terms used to search Cochrane

Search	Results
#1 (Distal Cholangiocarcinoma):ti,ab,kw	62
#2 (Cholangiocarcinoma):ti,ab,kw	958
#3 Bile duct neoplasms):ti,ab,kw	537
#4 Bile duct cancer):ti,ab,kw	1007
#5 (Minimally Invasive Pancreatoduodenectomy):ti,ab,kw	29
#6 (Laparoscopic Pancreatoduodenectomy):ti,ab,kw	48
#7 (Robot-Assisted Pancreatoduodenectomy):ti,ab,kw	15
#8 (Laparoscopic Whipple Procedure):ti,ab,kw	1
#9 (Robotic Whipple Procedure):ti,ab,kw	3
#10 (Open Pancreatoduodenectomy):ti,ab,kw	73
#11 (traditional Pancreatoduodenectomy):ti,ab,kw	10
#12 (Open Whipple Procedure):ti,ab,kw	11
#13 #1 OR #2 OR #3 OR #4	1674
#14 #5 OR #6 OR #7 OR #8 OR #9 OR #10 OR #11 OR #12	105
#15 #13 AND #14	9

Inclusion and exclusion criteria

Types of Study

For our research comparison of MIPD to OPD in patients with dCCA, we conducted a systematic review of relevant studies published in PubMed, Scopus, Cochrane, and Web of Science from 2003 to 2024, available in English. We meticulously screened and analyzed randomized clinical trials (RCTs) and cohort studies. This systematic review included studies that met the following inclusion criteria: RCT and cohort studies reporting on short-term and long-term outcomes in patients who underwent MIPD and OPD for dCCA. We excluded case-control studies, cross-sectional studies, case reports, case series, dissertations, book chapters, protocol articles, reviews, news articles, conference abstracts, letters to the editor, editorials, and comment publications. Furthermore, we excluded studies that did not clearly describe their operationalization, duplicates, and those for which we could not obtain the necessary data or receive a response from the original author via email.

Types of Participants

This study has specific participant selection criteria that include both sexes of patients over the age of 18. The focus is on patients diagnosed with dCCA who underwent MIPD or OPD. Studies that did not report intraoperative and postoperative outcomes or featuring patients that did not undergo MIPD or OPD as treatment, were excluded from the study.

Types of intervention

To be eligible for inclusion in this study, the selected research was evaluated for short-term and long-term outcomes after MIPD and OPD for dCCA. An MIPD includes robot-assisted and laparoscopic techniques. This review has a variety of participants to gain a better understanding of the intervention.

Outcomes

The outcomes to be evaluated after MIPD and OPD for dCCA include studies that report short-term outcomes such as operative time, intraoperative blood loss, length of hospital stays, postoperative 30-day morbidity and mortality, postoperative pain, and the time taken to return to regular activity or work. Long-term outcomes such as overall survival, disease-free interval, and recurrence rates are also measured. Studies that do not report outcomes in postoperative patients or patients with a different diagnosis were excluded.

Selection of Studies​​​​​

​Following an initial screening based on the title and abstract, two reviewers (NAW, KMN) independently selected trials for inclusion in this review using predetermined inclusion and exclusion criteria. This search used Rayyan (Rayyan Systems Inc., Cambridge, MA) to extract relevant data, and duplicates were filtered. Keywords were employed to highlight the search results via Rayyan [19]. We resolved disagreements about including studies by consensus and consultation with a third review author (ECM). 

Assessment of risk of bias in included studies

We conducted the evaluation of the data using the criteria outlined in Cochrane. To assess the quality of studies included in this systematic review, we will apply the Cochrane risk of bias (RoB) 2.0 tool [20], which examines potential bias in domains including selection, performance, detection, reporting, attrition, and other sources of bias, for RCTs. Additionally, for case-control studies included in the review, we employed the Newcastle-Ottawa scale (NOS) [21] to assess the RoB.

Two independent reviewers evaluated the RoB in each study, considering the specific criteria and guidelines provided by the respective tools. Any discrepancies between the reviewers were resolved through discussion or by consulting with a third, blinded reviewer as needed. The methodological components of the trials and case-control studies will be assessed as having a low, high, or unclear RoB in accordance with the Cochrane Handbook for Systematic Reviews of Interventions [22] and the NOS guidelines [21], respectively. Details of any down-or-upgrading of the quality of evidence are presented in the summary of findings, providing transparency and explanations for the assessment of bias in each study included. 

Statistical analysis 

Meta-analysis was performed using R Software version 023.09.1+494 (R Foundation for Statistical Computing, Vienna, AUT) to calculate the effect size [23]. Effect sizes were presented as mean differences with a 95% CI. The random‐effects model was used for pooling analysis to compensate for the heterogeneity of studies [23,24] statistics. In this regard, I2 ≥ 50% and ≥75% indicated substantial and considerable heterogeneity [24] study removal method to the subanalysis to assess whether any individual study exerted particular influence on the overall effect size [25,26]; p‐values < 0.05 were considered statistically significant.

Results

**Figure 1:**
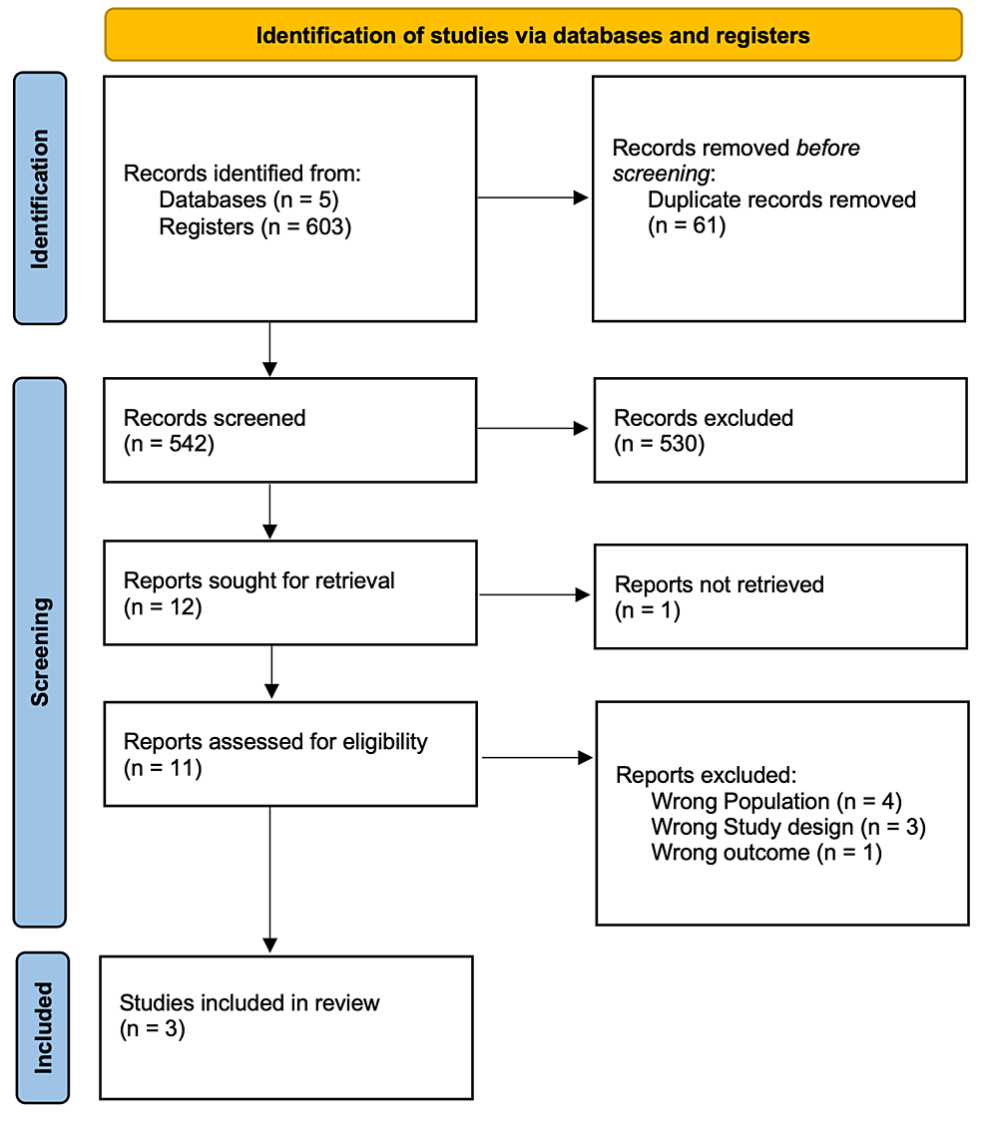
PRISMA flow diagram PRISMA: Preferred Reporting Items for Systematic Review and Meta-Analysis

Our initial search strategies across five databases for RCTs and cohort studies yielded 603 possible articles. After removing 61 duplicate articles, we screened based on title and abstract, excluding 530 articles. Subsequently, 12 were identified for retrieval. Among the remaining articles, one was not retrieved, and we assessed 11 full-text articles for eligibility, ultimately including three cohort studies in the review. All this is summarized in our PRISMA flow chart (Figure 1).

The RoB assessment utilized the NOS [21] for all three cohort studies. Our results (Table 6) show that all three selected studies were deemed to be of good quality. 

**Table 6:** Cohort studies assessed for RoB using the NOS RoB: Risk of bias, NOS: Newcastle-Ottawa scale for cohort studies Very good studies: 9 to 10 points; Good studies: 7 to 8 points; Satisfactory studies: 5 to 6 points; Unsatisfactory studies: 0 to 4 points. A study can receive a maximum of one star for each numbered item in the 'selection' and 'result' categories. A maximum of two stars can be given for comparability.

No.	Author/year	Study design	Selection	Comparability	Outcome/exposure	Total	Subjective evaluation
1	Shuai Xu et al., 2022 [23]	Cohort	★★★★	★	★★★	8	Good
2	Kim S et al., 2022 [24]	Cohort	★★★	★	★★★	7	Good
3	Uijterwijk et al., 2022 [25]	Cohort	★★★★	★	★★★	8	Good

The total sample size of the three included studies was 1349 patients, with 405 undergoing MIPDs and 944 undergoing OPDs. The participants were from various countries, including the Netherlands, Germany, Italy, Australia, Singapore, Korea, and China. Notably, one study was conducted exclusively in Korea, while another was carried out solely in China [21]​​​​​​ (Table 7). All three studies report similar findings regarding long-term outcomes. Uijterwijk et al. [23] and Kim et al. [24] suggest that MIPD is comparable to OPD in terms of long-term outcomes, while Xu et al. [25] indicates no significant differences in long-term survival between RPD and OPD groups. 

**Table 7:** General outcomes of the included studies MIPD: Minimally invasive pancreatoduodenectomy, OPD: Open pancreatoduodenectomy, PD: Pancreaduodenectomy, dCCA: Distal cholangiocarcinoma, RPD: Robotic pancreaticoduodenectomy

Author and year	Location	Study design	Intervention	Total sample	Results	General comments
Xu et al., 2022) [23]	China	Retrospective cohort-matched score	RPD	445	Robotic pancreaticoduodenectomy demonstrated better results in blood loss and length of hospital stay than OPD.	Robotic pancreaticoduodenectomy and OPD have similar outcomes to survival, with no significant changes in operative time or complications.
Kim et al., 2022 [24]	Korea	Retrospective cohort-matched score	MIPD	426	MIPD demonstrated lower blood loss and reduced hospital stay time. It also highlighted the lower number of complications and blood transfusions through this surgical procedure. Regarding the operation time, it was observed that OPD has a shorter operation time.	MIPD showed comparable postoperative complications and long-term oncological survival with OPD in the treatment of dCCA.
Uijterwijk et al., 2023 [25]	N/A	Retrospective cohort-matched score	MIPD	478	Minimally invasive pancreatoduodenectomy proved to have advantages over OPD in blood loss, mortality, transfusions, and complications.	It should have included the comorbidities of the patients for the selection of surgical procedures and as prognostic factors.

While each study focuses on different aspects of short-term outcomes, they all mention certain similarities. Uijterwijk et al. highlight the advantages of MIPD over OPD in terms of blood loss (300 mL vs. 500 mL) and surgical site infections. Xu et al. mention lower estimated blood loss (150 mL vs. 250 mL) and a shorter postoperative length of stay (12 vs. 15 days) in the RPD group compared to the OPD group. Kim et al. (2022) report more favorable results for MIPD in terms of blood loss (250 vs. 400 mL) and hospital stay (19.8 vs. 26.6 days) compared to OPD, but no significant difference in the major complications rate. While Xu et al. specifically mention no significant difference in operative time between the RPD and OPD groups, Kim et al. note that the OPD group had a more favorable result in terms of operation time compared to the MIPD group (398 vs. 457 minutes).

Meta-analysis results

The meta-analysis compared MIPD and OPD across three clinical outcomes: blood transfusion, complications, and mortality. No other variables were possible due to a lack of information. The analysis incorporated data from three studies, encompassing 1349 observations (405 for MIPD and 944 for OPD).

Blood Transfusion, Mortality, and Complications

**Figure 2:**
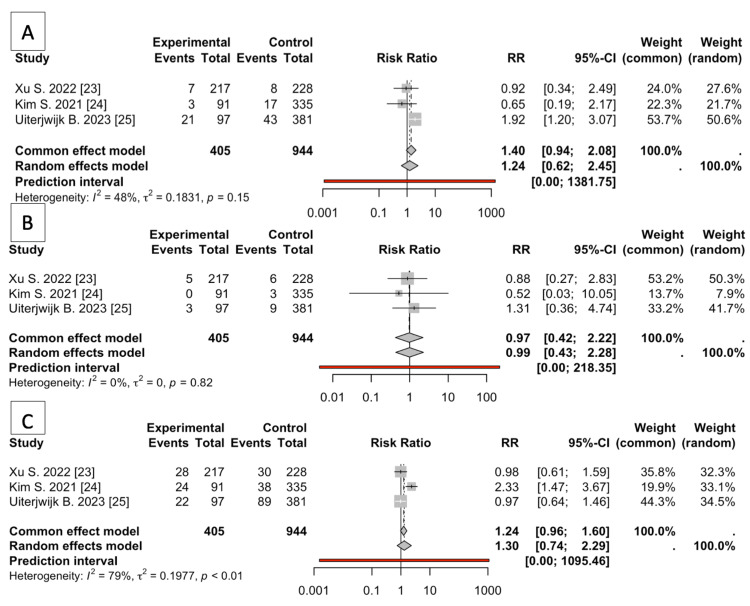
Forest plots A: The blood transfusion forest plot illustrates the RR of blood transfusion in three studies indicating substantial uncertainty in the effect estimate with moderate heterogeneity (I^2 = 47.7%). B: The mortality forest plot illustrates the RR of mortality in three studies indicating no significant difference in mortality between MIPD and OPD (p = 0.9893). Heterogeneity was negligible (I^2 = 0.0%). C: The complications forest plot illustrates the RR of complications in the same three studies. This model suggested a higher risk in the MIPD group compared to the OPD group although the results are statistically nonsignificant and there was substantial heterogeneity (I^2 = 79.0%). MIPD: Minimally invasive pancreatoduodenectomy, OPD: Open pancreatoduodenectomy

For blood transfusion, the random effects model reported an RR of 1.2372 (95% CI: 0.6239 to 2.4536) (Figure 2A). This does not reach statistical significance (p = 0.5423). The prediction interval was extremely wide (0.0011 to 1381.7468 ), indicating substantial uncertainty in the effect estimate. Heterogeneity was moderate (I^2 = 47.7%, tau^2 = 0.1831), although the test of heterogeneity was not significant (Q = 3.82, df = 2, p = 0.1480). The random effects models for mortality indicated an RR of 0.9943 (95% CI: 0.4328 to 2.2842). This model demonstrates no significant difference in mortality between MIPD and OPD (p = 0.9893) (Figure 2B). The prediction interval ranged from 0.0045 to 218.3503, suggesting high variability in potential outcomes. Heterogeneity was negligible (I^2 = 0.0%, tau^2 = 0), and the test for heterogeneity was non-significant (Q = 0.40, df = 2, p = 0.8181). In the analysis of complications, the random effects model estimated an RR of 1.3008 (95% CI: 0.7386 to 2.2907) (Figure 2C). This model suggested a higher risk in the MIPD group than in OPD, but these results were not statistically significant (p = 0.3624, respectively). The prediction interval was again very wide (0.0015 to 1095.4573), reflecting considerable uncertainty. There was substantial heterogeneity (I^2 = 79.0%, tau^2 = 0.1977), with a significant heterogeneity test (Q = 9.52, df = 2, p = 0.0086).

Publication Bias

**Figure 3:**
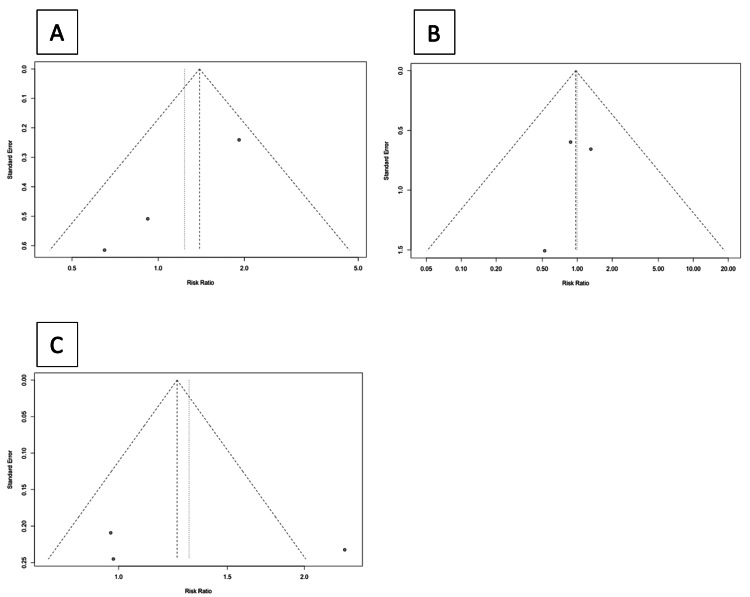
Funnel plots A: The funnel plot for blood transfusion assesses the publication bias in the meta-analysis of surgical interventions in patients with dCCA. The plot shows a symmetrical distribution of studies around the mean size, suggesting minimal publication bias in blood transfusion outcomes. The Egger test could not be performed due to the number of studies. B: The funnel plot for mortality assesses the publication bias in the meta-analysis of surgical interventions in patients with dCCA. The plot shows a symmetrical distribution of studies around the mean size, suggesting minimal publication bias in mortality outcomes. The Egger test could not be performed due to the number of studies. C: The funnel plot for complications assesses publication bias in the meta-analysis of surgical interventions in patients with dCCA. The plot shows an asymmetric distribution of studies around the mean size, indicating that smaller or non-significant studies might have been omitted concerning complication outcomes. dCCA: Distal cholangiocarcinoma

In this meta-analysis, the examination of publication bias reveals contrasting indications. The symmetric funnel plots for blood transfusion and mortality (Figure 3A-3B) suggest minimal publication bias in these outcomes. However, the asymmetry observed in the funnel plot for complications (Figure 3C) raises concerns about potential publication bias, indicating that smaller or non-significant studies might have been omitted. Due to the limited number of included studies, the inability to conduct an Egger test further constrains our ability to assess publication bias robustly. This limitation necessitates a cautious approach to interpreting the results, particularly for the outcome of complications. It underscores the need for larger studies or comprehensive meta-analyses to address the potential for publication bias adequately.

Discussion

The dCCA is a rare condition with a poor prognosis, and the only curative treatment is resection with negative margins [1]. Both the MIPD and OPD surgical techniques are available to achieve this goal, but there has not been a thorough comparison between these procedures. Therefore, a systematic review and meta-analysis were determined to be crucial to analyzing the diversity of evidence available to reach a common ground and choose the appropriate treatment regarding short- and long-term outcomes.

Our systematic review indicates that minimally invasive techniques may provide advantages over traditional open procedures in specific areas, such as reducing blood loss, postoperative length of stay, and surgical site infections, while maintaining comparable long-term outcomes and rates of major complications. However, upon comparing these findings with our meta-analysis, discrepancies emerge. The notion that MIPD can effectively reduce blood loss and the need for blood transfusions in dCCA cases remains inconclusive because the random effects model reported a relative risk of 1.2373 (95% CI: 0.6239 to 2.4536, p = 0.5423). There is also a need to look at the number of adverse events related to blood transfusion that are not counted as complications; further clarity on this topic will help prevent these [27]. Moreover, regarding complication rates and mortality, both our systematic review and meta-analysis suggest that the observed differences were not statistically significant. The meta-analysis for complications reported a p-value of 0.3624 (RR 1.30, 95% Cl: 0.74-2.29), while for mortality, the reported p-value was 0.9893 (RR 0.9943, 95% Cl: 0.4328-2.2842). 

Other meta-analyses comparing MIPD and OPD for pancreatic cancer, although not specifically focusing on dCCA, have shown differing results compared to ours. For instance, Wang et al. [26] reported a mean difference of 300.14 mL (95% CI: -400.11 to -200.17 mL, p = 0.00001) in estimated blood loss favoring MIPD, while Zhang et al. [28] reported a standard mean difference of 312 mL. These variations could be attributed to differences in sample size and article inclusion criteria; Zhang et al. included 22 retrospective studies with 6120 patients, whereas Wang et al. included 27 studies totaling 6909 patients. Interestingly, two studies included in our systematic review reported longer operation times, aligning with the outcomes reported in their meta-analyses. Regarding postoperative length of stay, our systematic review mirrors their findings, showing a shorter length of stay in the MIPD group. In terms of mortality and complications, our meta-analysis found no significant differences between the two procedures, consistent with the results reported in their meta-analyses. Several factors may contribute to the similar outcomes observed between both procedures. This could include the proficiency of surgical teams and the advancements in surgical techniques, as well as standardized postoperative care that leads to similar recovery trajectories, which collectively contribute to comparable results in terms of mortality, complications, and other key outcomes.

Another relevant study to contrast our findings would be the article by Kim et al. [29] who compared minimally invasive and open pancreatectomy for nonfunctioning pancreatic neuroendocrine tumors (NF-PNET). In the case of patients with a proximal location lesion, there was no significant difference in the average rates of postoperative complications. Obtaining similar findings as those in our review, such as average length of postoperative stay, did not significantly differ between the open group (20 days) and the minimally invasive surgery group (13 days) (p = 0.210). Also, a longer average operation time for minimally invasive surgery (512 min) than open procedure (346 min) was established with a p-value < 0.001, possibly related to the more complex skills needed. Furthermore, there was not a significant difference in the number of lymph node samples between the open group and the minimally invasive group (p = 0.804).

These findings suggest that MIPD may offer advantages over traditional open procedures in terms of postoperative length of stay and surgical site infections for patients with dCCA. The observed shorter postoperative length of stay associated with minimally invasive techniques may support the implementation of enhanced recovery after surgery (ERAS) protocols for dCCA patients undergoing pancreatoduodenectomy [30]. This may lead to enhanced patient experiences and satisfaction with a faster recovery, improved patient outcomes, and an earlier return to daily activities, as well as lower health care costs. However, it is essential to acknowledge the limitations of this study, which highlight areas for further investigation and underscore the need for caution in interpreting the results. 

Limitations

Our meta-analysis has several limitations that merit attention. First, the inclusion of only three studies limits the breadth of data, potentially affecting the generalizability of the findings. Second, substantial heterogeneity was observed, particularly in the complication’s outcome (I^2 = 79.0%), suggesting variability in patient demographics or surgical techniques. This heterogeneity introduces complexity in interpreting the pooled results. Furthermore, the prediction intervals across all outcomes were notably wide, reflecting significant uncertainty in the effect estimates. This suggests that the true effects of MIPD versus OPD could vary extensively across different clinical settings. Our analysis also lacked subgroups and sensitivity analyses, crucial in understanding the influence of different patient or study characteristics on the outcomes.

Additionally, the potential for publication bias inherent in meta-analyses, especially with a limited number of studies, cannot be ignored. Such bias could skew the interpretation of the results. Finally, the quality and RoB within the included studies were not explicitly addressed, which is a critical factor affecting the reliability of meta-analytic conclusions.

Future research should further validate the clinical relevance and assess the efficacy of minimally invasive techniques for dCCA. This entails expanding the scope of outcomes to include long-term survival, quality of life, and hospital length of stay, which will provide a more comprehensive understanding of the comparative effectiveness of MIPD versus OPD. Additionally, larger-scale studies incorporating individual patient data are necessary to enhance the robustness and external validity of the findings, allowing for adjustments for confounding factors and a better understanding of their impact on different patient subgroups.

## Conclusions

Our systematic review and meta-analysis filled a crucial knowledge gap by comparing the effectiveness of MIPD versus OPD in patients with dCCA. Despite the significant morbidity rates associated with both procedures and the aggressive nature of dCCA, our analysis uncovered key insights. While our systematic review hints at potential benefits of MIPD in terms of postoperative length of stay, surgical site infections, and reduced blood loss compared to OPD, our meta-analysis found no statistically significant differences in outcomes like blood transfusions, complications, or mortality between the two procedures. These findings align with existing meta-analyses on pancreatic cancer, indicating potential influences from factors such as surgical expertise, postoperative care quality, and surgical advancements.
